# Investigation of beam splitter in a zero-refractive-index photonic crystal at the frequency of Dirac-like point

**DOI:** 10.1038/s41598-017-10056-z

**Published:** 2017-08-29

**Authors:** Pingping Qiu, Weibin Qiu, Zhili Lin, Houbo Chen, Junbo Ren, Jia-Xian Wang, Qiang Kan, Jiao-Qing Pan

**Affiliations:** 1 Fujian Key Laboratory of Light Propagation and Transformation, College of Information Science and Engineering, Huaqiao University, Xiamen, 361021 China; 2 College of Materials Science and Opto-Electronic Technology, University of Chinese Academy of Sciences, Beijing, 100086 China; 3 Institute of Semiconductors, Chinese Academy of Sciences, Beijing, 100086 China

## Abstract

The Dirac-like cone dispersion of the photonic crystal induced by the three-fold accidental degeneracy at the Brillouin center is calculated in this paper. Such photonic crystals can be mapped to zero-refractive-index materials at the vicinity of the Dirac-like point frequency, and utilized to construct beam splitter of high transmission efficiency. The splitting ratio is studied as a function of the position of the input/output waveguides. Furthermore, variant beam splitters with asymmetric structures, bulk defects, and some certain bending angles are numerically simulated. Finally, we show that 1 × 2 to 1 × N beam splitting can be realized with high transmission efficiency in such a zero-refractive-index photonic crystal at the frequency of Dirac-like point. The proposed structure could be a fundamental component of the high density photonic integrated circuit technique.

## Introduction

In recent years, metamaterials with zero-refractive-index have attracted considerable attention due to their intriguing properties and novel applications. Such as realization of total transmission and reflection^1, 2^, directional emission^3, 4^, supercoupling^5, 6^, unidirectional transmission^7^, and so on^8–10^. However, experimental demonstration of zero-refractive-index metamaterials (ZIM) typically involved metals working around their plasma frequencies^11, 12^, which suffered significant Ohmic losses and impedance mismatch. Unlike the conventional ZIM comprising metallic elements, the appropriately designed all-dielectric photonic crystals (PCs) with Dirac-like cone dispersion could be mapped to an impedance-matched ZIM with extremely low loss at the vicinity of the Dirac-like point frequency, which improved the functionality of the ZIM-based devices in a large extent^13–15^.

The Dirac-like cones of the PCs are a consequence of the three-fold accidental degeneracy at the Brillouin zone (BZ) center^16^. By utilizing effective medium theory, it has been proven that both the effective permittivity and permeability of the PCs with the Dirac-like cone formed by two dipolar modes and a single monopole mode are simultaneously zero at the Dirac-like point frequency^13, 17^. Huang *et al*. theoretically investigated the first all-dielectric ZIM constructed by a two-dimensional (2D) PC with a square lattice and experimentally demonstrated that such ZIM realized the cloaking effect in microwave frequency^13^. In 2013, Moira *et al*. experimentally proved an impedance-matched ZIM at optical frequencies, which exhibited a nearly isotropic low-index response for transverse magnetic (TM) polarized light, resulting in angular selectivity of transmission and directive emission from quantum dots positioned within the material^15^. In 2015, Li *et al*. reported the first on-chip integrated ZIM at the optical regime, which enabled the direct implementation of zero-index effect on a chip^14^. Furthermore, Hajian *et al*. claimed that by using all-dielectric zero-refractive-index photonic crystals (ZIPC), a considerable transmission enhancement was achieved in a waveguide system due to the well-matched impedance near the Dirac frequency^18^. Some other interesting phenomena and applications including unidirectional transmission^19^, focusing lens^20^, tunneling effect^21^, and so on^22–25^, were also studied by using the intendedly designed ZIPC.

In this work, we propose a 2D all-dielectric PC composed of an array of dielectric cylinders in air with a honeycomb lattice. By appropriate design of the geometry parameters of PC, a Dirac-like cone formed by two dipolar modes and a single monopole mode can be realized at the BZ center, which indicates the zero-index characteristic. Further, beam splitter constructed by the ZIPC, which is completely different from the conventional beam splitter such as multiple mode interferometer (MMI) in mechanism, is numerically investigated. By optimizing the impedance and the input/out waveguide width, the transmission efficiency close to 100% can be obtained. And we also study the splitting behavior of the splitter with a defect or a certain bending angle. Eventually, we show that such a beam splitter can realize 1 × 3, 1 × 4…1 × N splitting with high transmission efficiency.

## Results and Discussion

**Figure 1:**
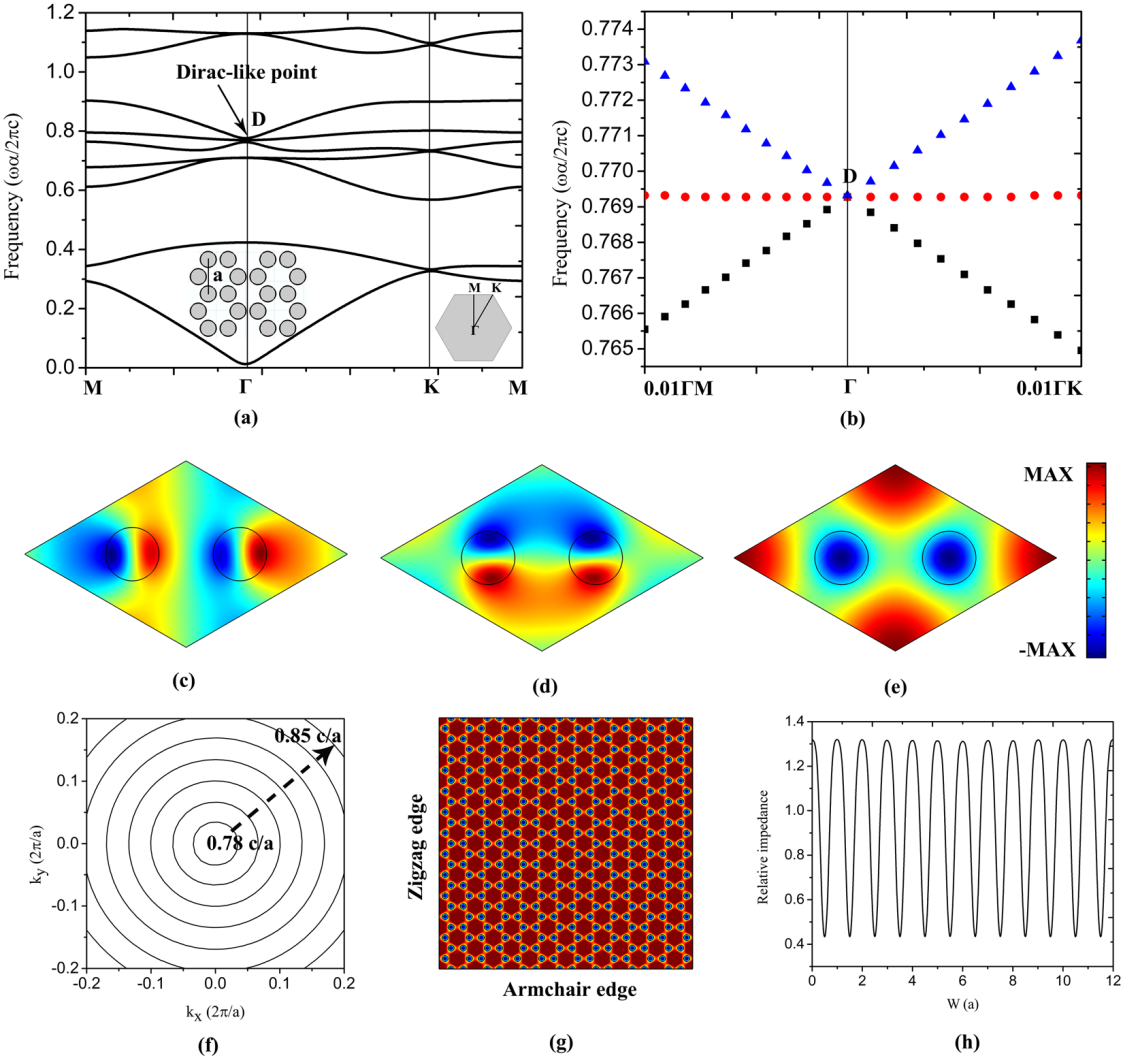
The photonic properties of ZIPC. (**a**) Band structure of the PC, the insets are schematic structure of the PC with a honeycomb lattice, BZ and *k* path for the honeycomb unit cell respectively. (**b**) Enlarged view of the band structure near the Dirac-like point. (**c**–**e**) electric field distributions of the three degenerate eigenstates at the Dirac-like point *D*, the field profiles in (**c**) and (**d**) are dipolar modes, and (**e**) is monopole mode. (**f**) Isofrequency contours of PC. (**g**) Eigen electric field distribution of the bulk PC with zigzag and armchair edges. (**h**) Relative impedance with respect to the zigzag edge of the bulk PC. *a* is the lattice constant, *r* = 0.1433*a* is the radius of the pillars.

The PC structure under consideration is an array of dielectric cylinders in air with a honeycomb lattice, as shown in the inset of Fig. 1(a). *a* is the lattice constant, the radius of the cylinders *r* = 0.1433*a*. The inset in the right corner of Fig. 1(a) denotes the BZ and *k* path for the honeycomb unit cell. The relative permittivity and permeability are set as *ε*_*r*_ = 12 and *μ*_*r*_ = 1, which is close to the parameters of silicon material used in semiconductor electronics. The band structures of the PC are calculated for TM polarization with the electric field along the axis of the cylinders.

The band structure of the all-dielectric PC with *r* = 0.1433*a* is plotted in Fig. 1(a), where we can see that two bands linearly intersect with an additional flat band at the BZ center, resulting in a three-fold degenerate point marked with “*D*”, which is the so-called Dirac-like point^26^. The Dirac-like point with a frequency of 0.7693 *c*/*a* (*c* is the speed of light) is composed of two dipolar modes (shown in Fig. 1(c) and (d)) and a single monopole mode (shown in Fig. 1(e)). It should be noted that the Dirac-like cone dispersion relations do not exist with other geometrical parameters in our PC structure, which means the Dirac-like cone is not a necessary result of the lattice symmetry but an accidental degeneracy achieved when appropriate geometrical and material parameters are designed. To have a more clear view of the Dirac-like cone dispersion, we zoom in the band structure near the BZ center as shown in Fig. 1(b), where two branches with linear dispersions intersect at the degenerate point *D*, through which point there is a another flat band resulting in the Dirac-like cone. In the vicinity of the Dirac-like point, the magnitude of the wave vector *k* in the PC is much smaller than that in air, which indicates that the effective refractive index of the PC *n*_*eff*_ is very small and close to zero due to the direct proportional relationship between *n*_*eff*_ and *k*. Therefore, the designed PC structure can model a zero-refractive-index medium. Like the ZIMs, when a beam of light impinges to a PC with a frequency of the Dirac-like point, it experiences a zero group velocity and an infinite phase velocity for the duration in the PC, which, in other words, there is no phase variation in the PC (see supplementary information). Light with a planar wave front impinges the PC with the zigzag edge as both the input and output interface with the free space, planar wave front is obtained at the output surface, though the amplitude of the light field is not uniform along the output edge. If the input/output beams are contained by waveguides, the whole configuration becomes the beam splitter/combiner. For the sake of simplicity, we only consider the splitter in this paper.

Furthermore, one can see from the nearly circular isofrequency contours shown in Fig. 1(f) that the PC is essentially isotropic around the Dirac-like point, which indicates our designed PC maintains a zero-refractive-index in almost all transmission direction as long as a full PC structure is ensured. The relative impedance is defined as the ratio between the electric field along the *z* direction and the magnetic field along the *y* direction, i.e. *Z*_*e*_ = −*E*_*z*_/(*H*_*y*_ *Z*_0_), where *Z*_0_ is the impedance of the free space^27^. Figure 1(h) plots the relative impedance along the zigzag interface when the bulk behavior of PC (shown in Fig. 1(g)) is excited at the Dirac frequency of 0.7693 *c*/*a*, where we find that the surface impedance of the PC is location-dependent, which means we can modulate the coupling efficiency via tuning the position of input/output waveguides connected to PC.

**Figure 2:**
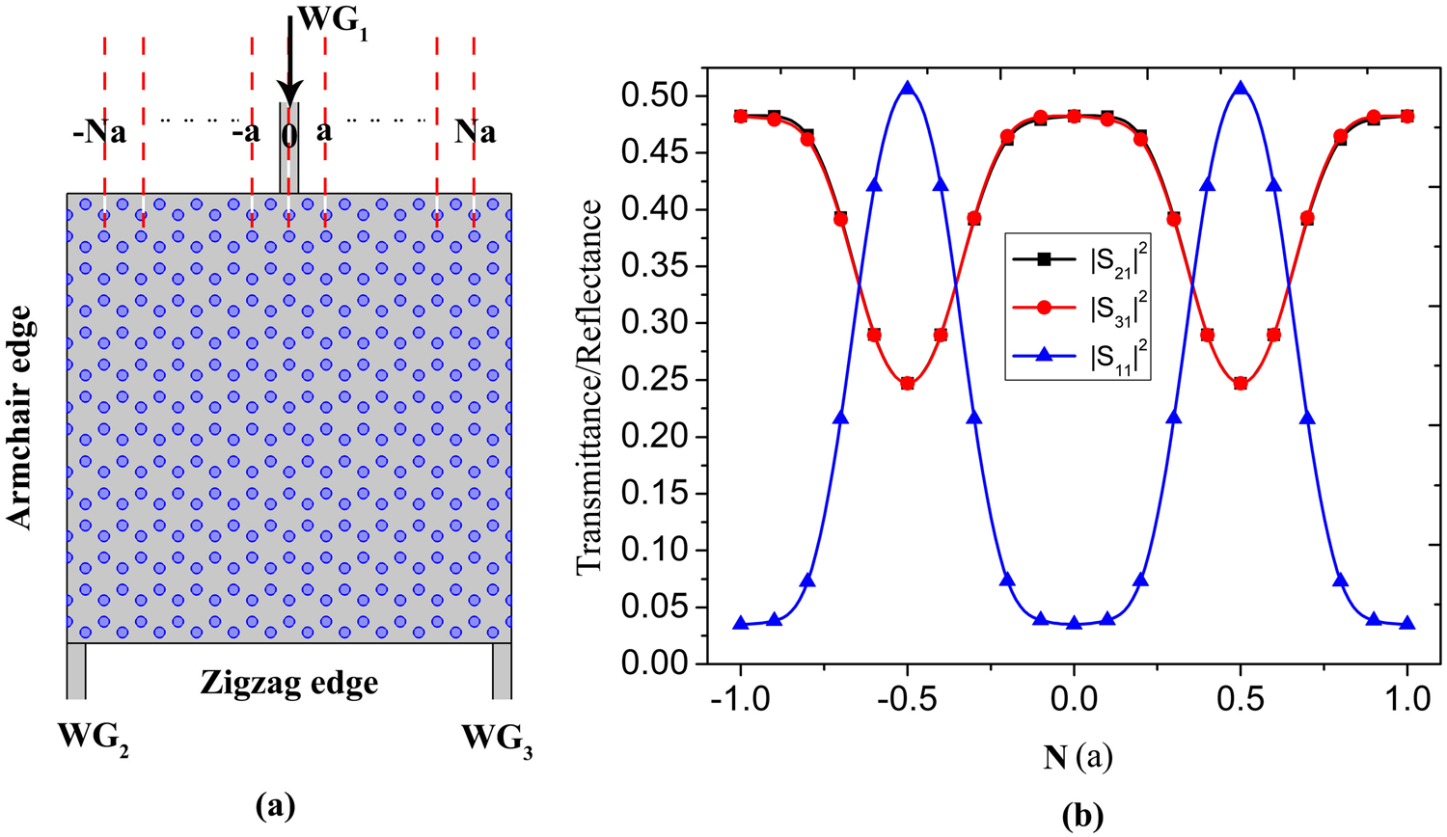
(**a**) The schematic structure of the beam splitter constructed by ZIPC with armchair and zigzag edges. (**b**) The transmittance and reflectance spectrums of the beam splitter with the input waveguide *WG*_1_.at different locations (from −*a* to *a*). The width of the input and output waveguides is set as 0.5*a*.

At the Dirac-like point, the PC exhibits zero effective permittivity and permeability, leading to an impedance-matched ZIM^15^. Light inside such ZIPC experiences no spatial phase change and extremely large phase velocity, therefore, by appropriate design of input and output waveguides, beam splitter is expectable. In this work, the ZIPC with armchair and zigzag edges is used to form the beam splitter as shown in Fig. 2(a). Firstly, we keep the location of the output waveguides (at the both sides of the output surface) constant, varying the location of the input waveguide. A plane wave with the Dirac-like point frequency of 0.7693*c*/*a* is normally incident from the input waveguide *WG*_1_. Figure 2(b) displays the transmittance/reflectance spectra of the ZIPC beam splitter, one can see that the transmittance of the two ports always equal regardless of the position of the input waveguide, and exhibits a periodic variation, which is due to the periodic impedance along the input surface. The highest transmission larger than 96% is achieved by tuning the position of *WG*_1_. Furthermore, in order to get a more higher transmission efficiency, both the effective surface impedance and the eigen mode matching between the input and output surface should be satisfied (see supplementary information). However, unlike the ZIM beam splitter cases, where both the effective surface impedance and the light field including the amplitude and the phase are uniformly distribution on both the input and output interfaces of the ZIM, the effective surface impedance and the eigen light field are position dependent and periodically distributed along the edge of the photonic crystal, which is governed by the Bloch theorem, and shown in Fig. 1(h) and the supplementary information.

**Figure 3:**
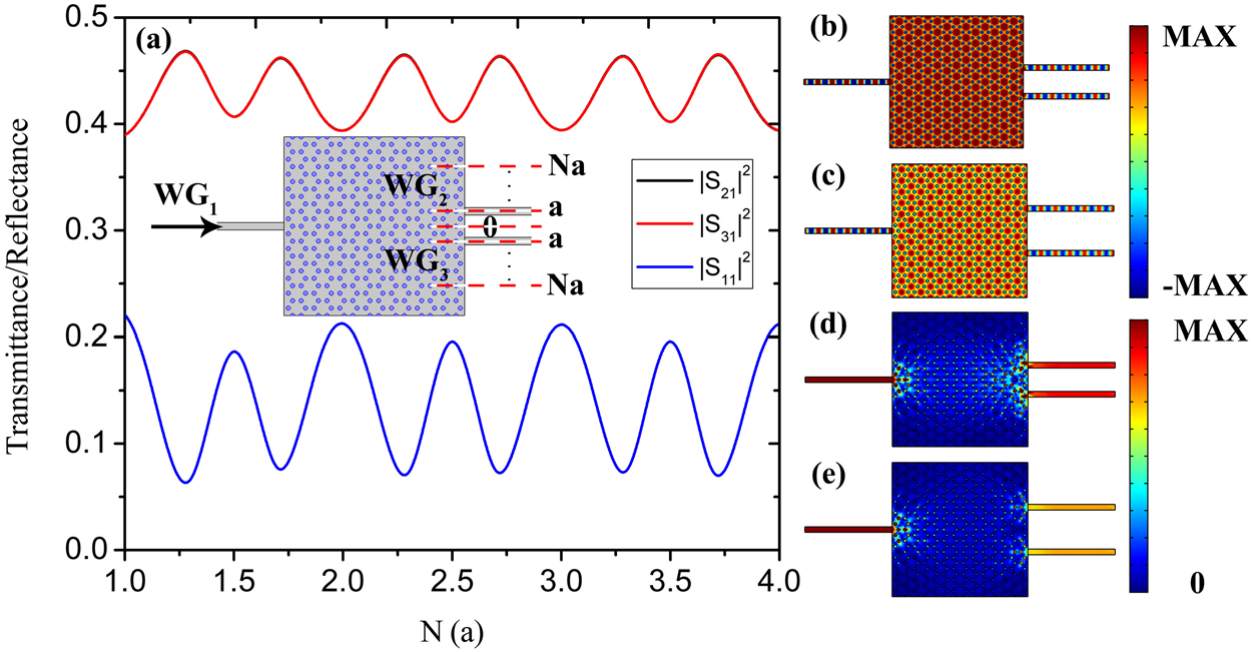
(**a**) The transmittance and reflectance spectrums of the beam splitter with the two output waveguides *WG*_1_, *WG*_2_ located from *a* to 4*a*, the inset is schematic structure of the beam splitter. (**b**–**c**) The electric field distributions of the beam splitter with the highest transmission and reflection respectively. (**d**–**e**) The corresponding energy flux density distributions.

Since we have a clear understanding about the location of input waveguide towards the beam splitter, further investigation about the output waveguides can be implemented. As illustrated in the inset of Fig. 3(a), we keep the *WG*_1_ at the center of input surface constant, then varying *WG*_2_ and *WG*_3_ from *a* to *Na* simultaneously. Figure 3(a) plots the transmittance/reflectance spectrums of beam splitter with *WG*_2_ and *WG*_3_ tuned from *a* to 4*a*, where we found that as long as the output waveguides are symmetric with the input waveguide, an equal transmission can be realized through output waveguides. By optimizing the impedance, transmission efficiency larger than 96% is obtained. Figure 3(b) and (c) display the electric field distributions at the transmission peak and dip respectively where we can see the uniform field distributions which is also the characteristic of ZIPC. The corresponding energy flux density distributions are shown in Fig. 3(d) and (e) which reveal the well-splitting ability and proportionality.

**Figure 4:**
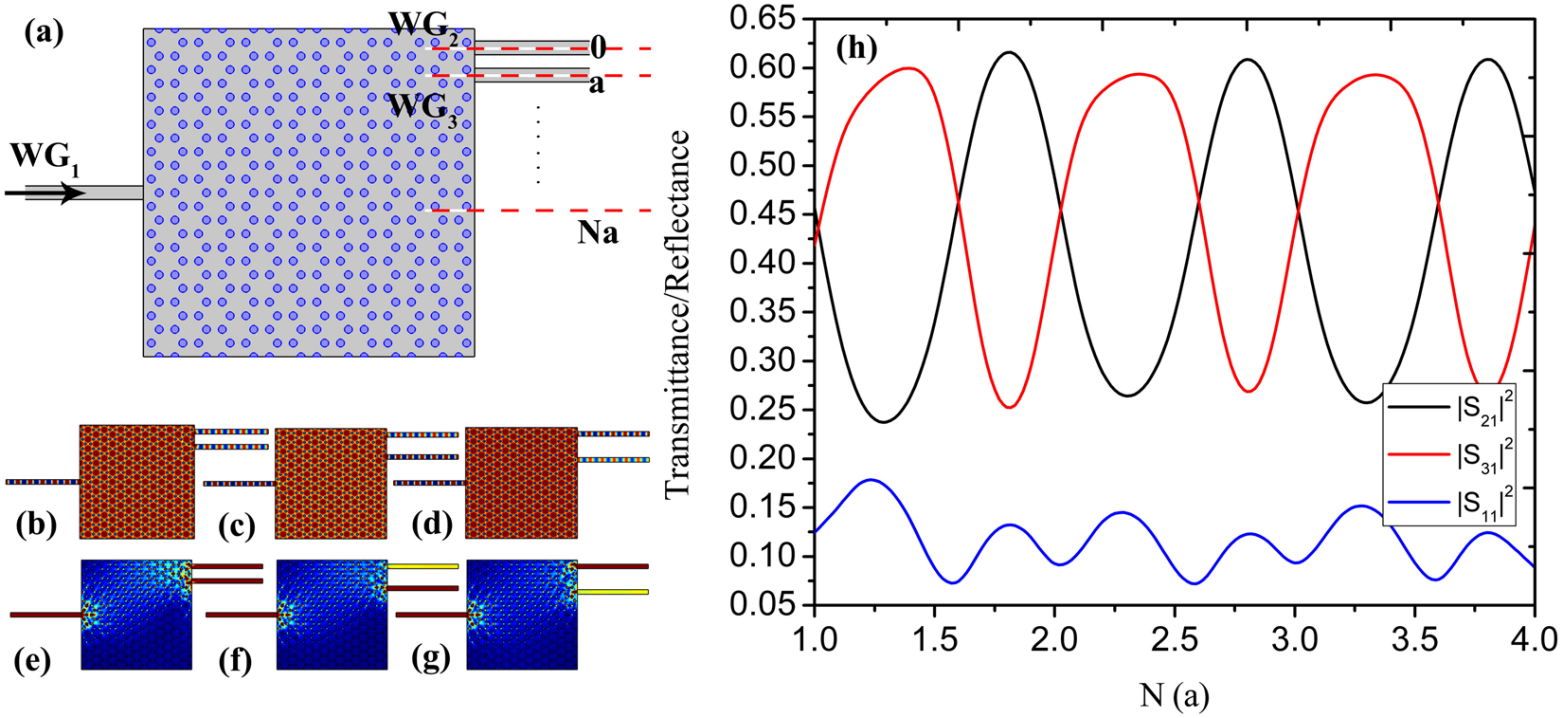
(**a**) The schematic structure of the beam splitter with *WG*_2_ located at 0, *WG*_3_ located from *a* to 4*a*; (**b**–**d**) the electric field distributions of the beam splitter with the equal output, the highest |S_31_|^2^ and the highest |S_21_|^2^. (**e**–**g**) The corresponding energy flux density distributions. (**h**) The transmittance and reflectance spectrums of the beam splitter shown in (**a**).

We know that the symmetric location of the output waveguides can lead to an equal splitting. Now, we keep *WG*_2_ constant, varying *WG*_3_ from *a* to *Na* as shown in Fig. 4(a). Figure 4(h) plots the transmittance/reflectance spectrums with *WG*_3_ tuned from *a* to 4*a*, where we can see the transmittance/reflectance spectrums are periodically varied. By modifying the position of *WG*_3_, one can obtain the equal transmission of 46%, the highest |S_21_|^2^ of 61.6% or the highest |S_31_|^2^ of 60%, which provides an effective way to tune the splitting ratio in a certain range. Noted that the reflectance is generally around 10%, which means a high transmission efficiency of 90%. Figure 4(b–d) display the electric field distributions of the beam splitter with equal splitting, the highest |S_31_|^2^ and the highest |S_21_|^2^ respectively, and Fig. 4(e–g) are the corresponding energy flux density distributions.

Further optimization shows that a total transmission of 100% is obtainable when both the effective surface impedance matching and mode matching conditions are satisfied, where the total width of the output waveguides equals to the input waveguide, which equals to a period of the photonic crystal, to ensure the duplication of the input optical mode at the output surface (see supplementary information). Of course, according to Bloch theorem, the output waveguides could locate at different unit cells of the PC. Also, the width of each output waveguide is not necessarily identical, if the even splitting ratio is not concerned.

**Figure 5:**
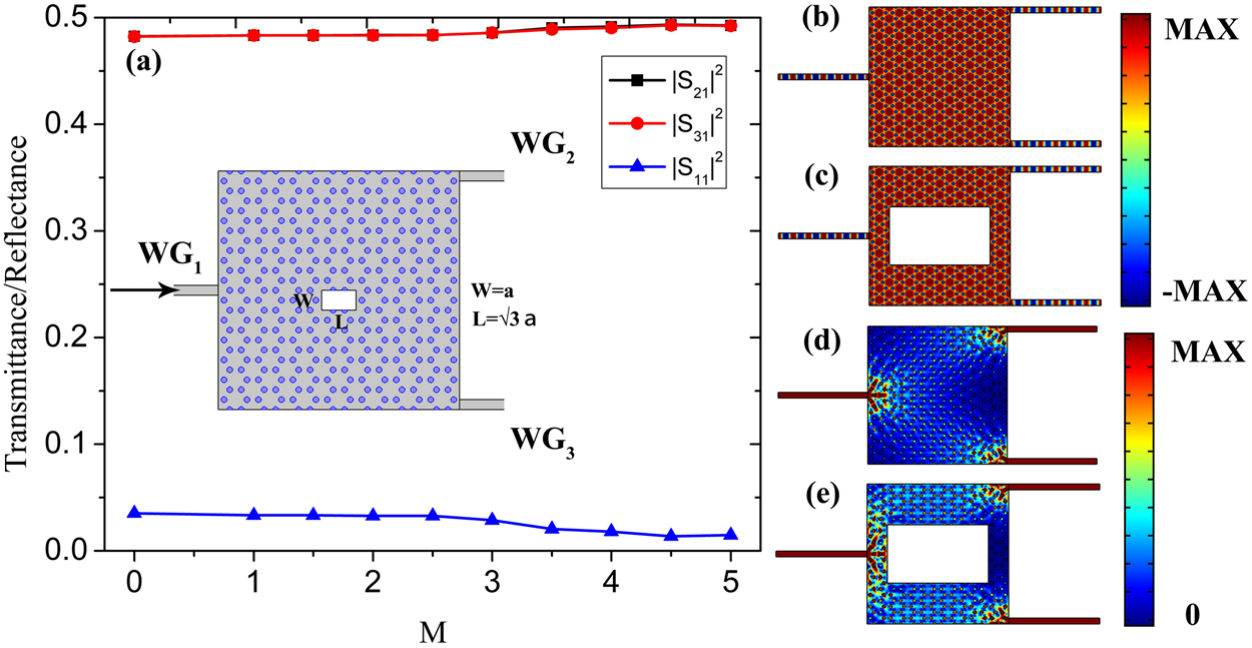
(**a**) The transmittance and the reflectance spectrums of the beam splitter with a M(*W*x*L*) defect (M = 1, 1.5, 2…4), the inset is the schematic structure of the beam splitter. (**b**,**c**) The electric field distributions of the beam splitter with no defect and a 5(*W* × *L*) defect. (**d**,**e**) The corresponding energy flux density distributions. *W* = *a*, *L* = √3*a*.

It has been demonstrated that there exists cloaking effect in the ZIPC due to the unique scattering properties of ZIM^13^. Therefore, similar phenomena are expectable in the ZIPC beam splitter by appropriate design of the defect. The inset in Fig. 5(a) illustrates the schematic structure of the beam splitter with a M(*W* × *L*) defect (M = 1, 1.5, 2…4). Noted that all these defects maintain the armchair and zigzag edges. The corresponding transmittance/reflectance spectrums of the defective beam splitter are displayed in Fig. 5(a), where we can find that the defects rarely influence the beam splitter, transmission wave equally outputs from two output waveguides with high transmission efficiency of over 96%. This also can be confirmed from the electric field distributions and energy flux density distributions shown in Fig. 5(b–e), which exhibits the uniform field distributions and equally split energy flux density with or without a defect.

**Figure 6:**
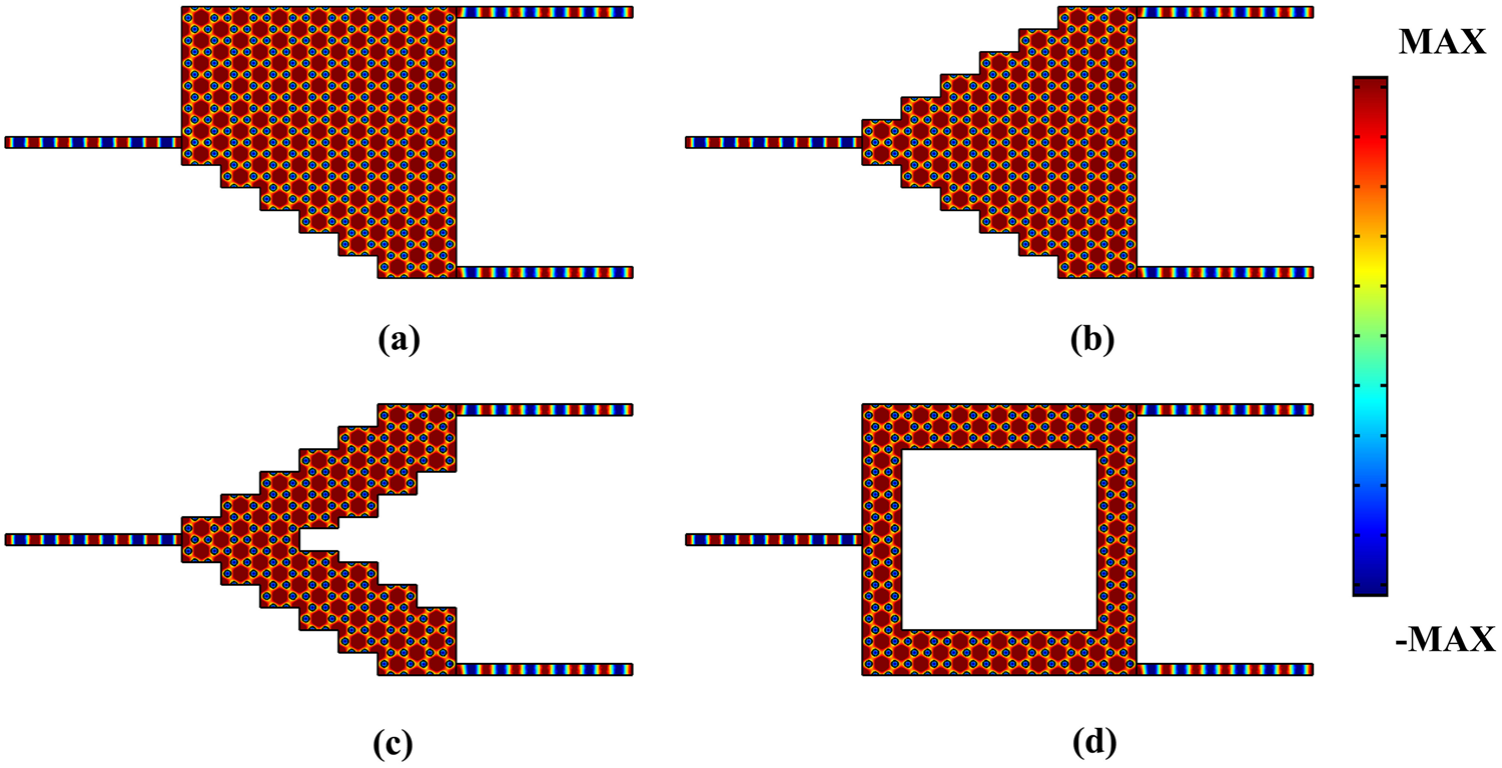
The electric field distributions of the beam splitter: (**a**) with asymmetrical ladder-like edge; (**b**) with symmetrical ladder-like edge; (**c**) with double symmetrical ladder-like edge; (**d**) with a large defect (8*a* × 5√3*a*).

In fact, the proposed ZIPC beam splitter possesses extraordinary impedance matching to free space and to standard optical waveguides only if it preserves the armchair and zigzag edges. This is significantly distinct from the multimode interferometer (MMI), where the beam splitting properties is rooted from the self-imaging effect of the input waveguide in the multiple mode waveguide region^28, 29^. Figure 6 displays electric field distributions of various kinds of defective beam splitter formed by the ZIPC, such as the beam splitter with asymmetrical ladder-like defect (Fig. 6(a)), symmetrical ladder-like defect (Fig. 6(b)) etc. It still works even with a couple of periods (Fig. 6(d)).

**Figure 7:**
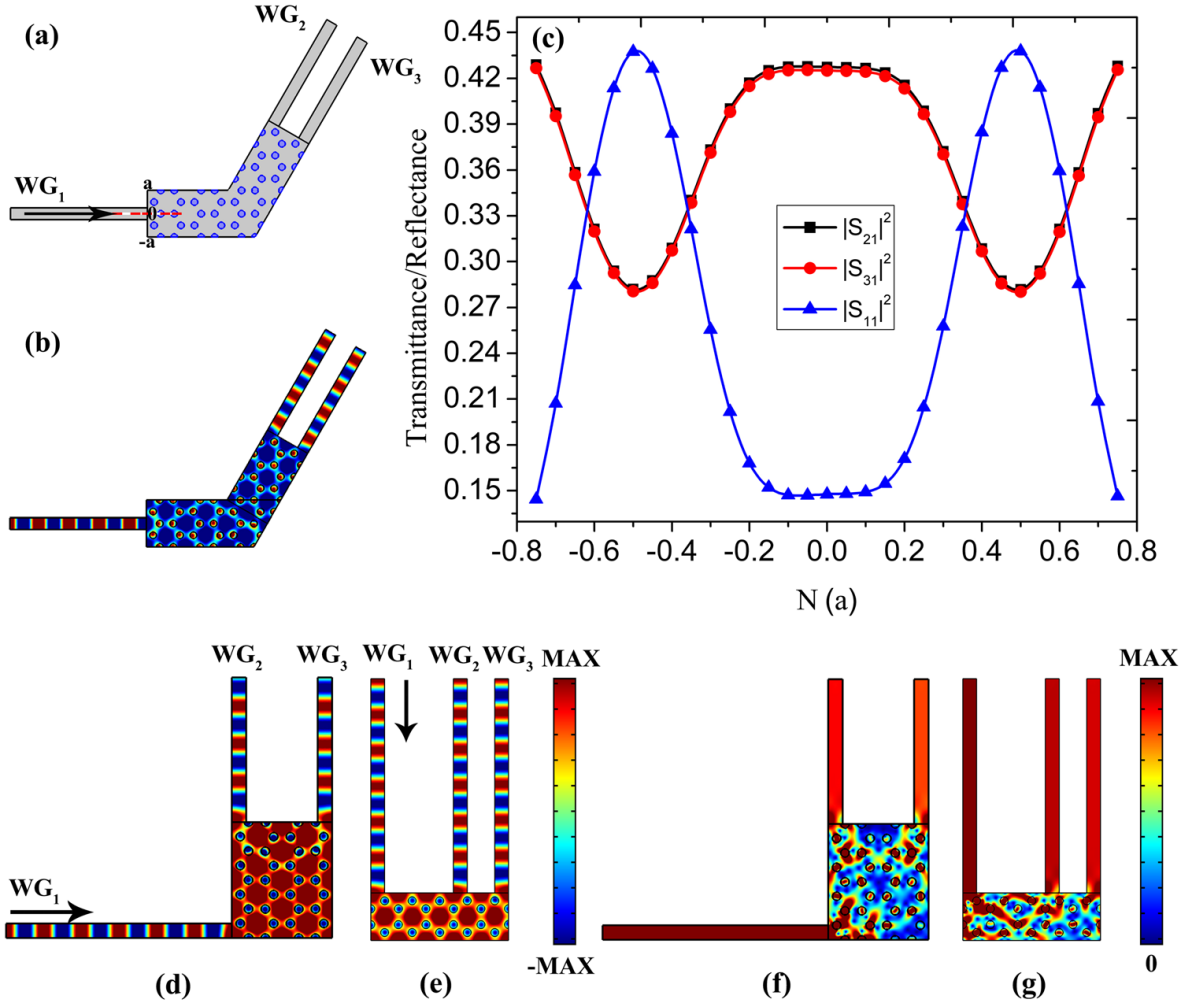
ZIPC beam splitters with a bending angle. (**a**) The schematic structure of the beam splitter with a bending angle of 30°. (**b**) The corresponding electric field distribution with *WG*_1_ positioned at 0. (**c**) The transmittance and reflectance spectrums of the bending beam splitter with input waveguide *WG*_1_ located from −0.75*a* to 0.75*a*. (**d**,**e**) The electric field distributions of the beam splitter with a bending angle of 90° and 180° respectively. (**f**,**g**) The corresponding energy flux density distributions. The width of the input and output waveguides is set as 0.5*a*.

At the Dirac-like point, electromagnetic waves propagate with infinite phase velocity and experience no spatial phase variation inside the ZIPC, and thus such a ZIPC beam splitter can still works with a certain bending angle to ensure the zigzag interface of the output, albeit the isofrequency contour shown in Fig. 1(f) is isotropic. Figure 7(a) plots the beam splitter with a bending angle of 30°, a plane wave with the Dirac-like point frequency incident from *WG*_1_, then transmits through the bending, eventually splits equally into *WG*_2_ and *WG*_3_. Figure 7(c) is the transmittance/reflectance spectrums of the beam splitter, where we can see that by tuning the location of input waveguide, transmission efficiency as high as 85% can be achieved. Figure 7(b) is the electric field distribution when the input waveguide located at the center of entrance surface, where a uniform field distribution with no phase change is observable. As we have explained that the ZIPC with good impedance matching to free space and to standard optical waveguides can realize arbitrary bending splitters under appropriate design of the input and output waveguides, Fig. 7(d,e) display electric field distributions of beam splitter with a bending angle of 90°, 180°, where uniform field distributions and well-splitting ability can be seen. A plane wave with Dirac-like point frequency incident from *WG*_1_, then propagates through ZIPC, finally splits equally into *WG*_2_ and *WG*_3_ without phase change. Figure 7(f,g) plot the corresponding energy flux density distributions.

**Figure 8:**
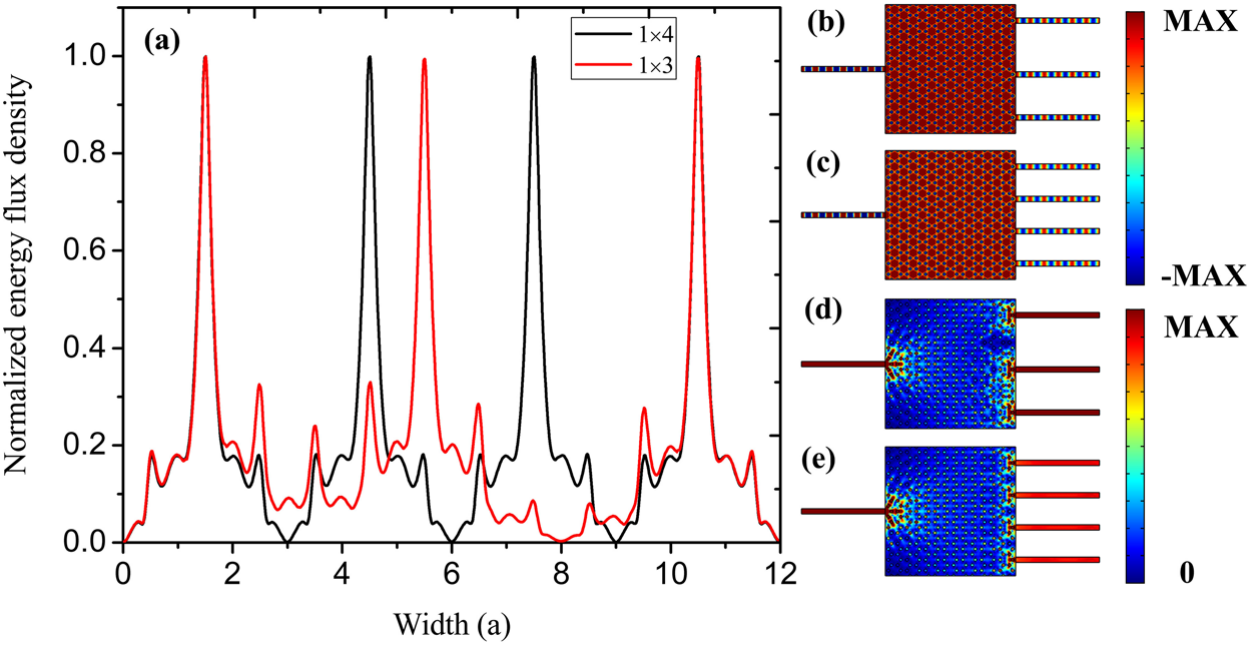
(**a**) The normalized energy flux density distributions of 1 × 3 and 1 × 4 beam splitter near the output waveguides. (**b**,**c**) The electric field distributions of the 1 × 3 and 1 × 4 beam splitter. (**d**,**e**) The corresponding energy flux density distributions.

Finally, we show that such a ZIPC can be utilized to achieve 1 × 3, 1 × 4…1 × N beam splitter considering the exceptional impedance matching to free space and optical waveguides. Figure 8(a) depicts the normalized energy flux density of 1 × 3 and 1 × 4 beam splitter near the output surface, where we can see the distinguished splitting ability. We also can see the uniform field distributions and equally split energy flux density distributions from Fig. 8(b–e), which reveals that such a ZIPC can couple energy into any number of output waveguides evenly with little impedance mismatch, resulting in a 1xN beam splitter. It is worth to point out that the beam splitting effect is completed within a whole period of the photonic crystal, which suggests a much more compact footprint than the configuration of MMI.

## Conclusion

In conclusion, we proposed an all-dielectric PC composed of an array of silicon pillars in air with a honeycomb lattice to create a Dirac-like cone at the BZ center. The Dirac-like cone is formed by two dipolar modes and a single monopole mode. Near the Dirac-like point, PC can be mapped to ZIM with good impedance matching to free space and optical waveguides. By using such a ZIPC, beam splitters with transmission efficiency close to 100% are achieved. Furthermore, we numerically simulated that ZIPC beam splitter can still work with high efficiency after a certain bending angle. At last, we show that such a ZIPC beam splitter can realize any number of beam splitting.

## Methods

The COMSOL Multiphysics, RF module, commercial software based on finite element method (FEM), was used for the simulations throughout this paper. The band structure of the PC was obtained by scanning the eigen frequency in the reduced Brillouin zone with the eigenfrency solver. The beam splitting properties of the proposed devices were got by using the frequency domain solver of the COMSOL Multiphysics, RF module.

## Electronic supplementary material

Supplementary Information
