# Supplementary material for: Investigation of beam splitter in a zero-refractive-index photonic crystal at the frequency of Dirac-like point
Source: Sci Rep. 2017 Aug 29;7:9588. doi: 10.1038/s41598-017-10056-z (PMC5574982; doi:10.1038/s41598-017-10056-z)
Supplement: Supplementary file 1 — Supplementary Information [file 41598_2017_10056_MOESM1_ESM.pdf]

Supplementary Information

Investigation of beam splitter in a zero-refractive-index photonic crystal at the frequency of Dirac-like point

Pingping Qiu[1], Weibin Qiu[1,*], Zhili Lin[1], Houbo Chen[1], Junbo Ren[1], Jia-Xian Wang[1], Qiang Kan[2,3], and Jiao-Qing Pan[2,3]

[1]Fujian Key Laboratory of Light Propagation and Transformation, College of Information Science and Engineering, Huaqiao University, Xiamen 361021, China;
[2]College of Materials Science and Opto-Electronic Technology, University of Chinese Academy of Sciences, Beijing 100086, China;
[3]Institute of Semiconductors, Chinese Academy of Sciences, Beijing 100086, China;
*Correspondence: wbqiu@hqu.edu.cn

1. Beam splitting effect of the zero index material (ZIM)

It is well known that when a beam of light impinges to a ZIM with a finite thickness much larger than the wavelength in the free space, it experiences a zero group velocity and an infinite phase velocity for the duration in the ZIM, which, in other words, there is no phase variation in the ZIM[S1]. Also, according to Snell's law, the output wave front is parallel to the surface of the output and immunes to the input surface of the ZIM[S2]. Fig. S1(a) and Fig. S1(b) indicate the maps of both the amplitude and the phase distribution of the light field. It is evident that both amplitude and the phase keep uniform at the output surface of the ZIM.

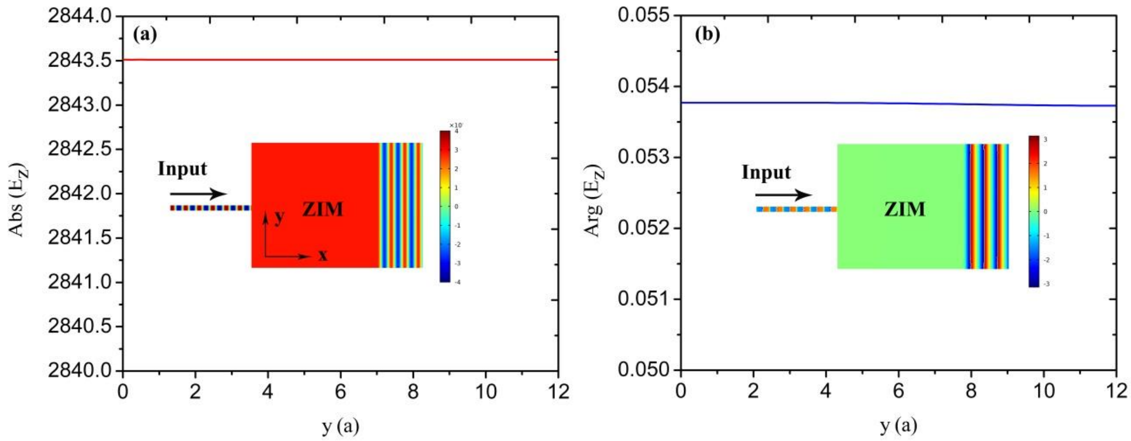

Figure S1. (a) Amplitude and (b) phase distributions at the exit surface of an actual ZIM with ε_r = 0.0001, μ_r = 0.0001. The insets are the corresponding electric field and phase distributions excited with a plane wave incident from the left input waveguide respectively.

2. Optimization of the transmission and the splitting ratio of the splitter consisted by ZIM

If both the input and output beams are contained by waveguides, the beam (power) splitters/combiners can be realized. This is the main mechanism of the beam splitters/combiners. For the sake of simplicity, we only discuss the splitters in this article. The insets of the Fig. S1(a) and Fig. S1(b) demonstrate the

schematic of 1X2 splitters. It should be noted that the positions of all the input and output waveguides do not influence the performance of the beam splitter due to the uniform distribution of both the amplitude and phase of the light on both the input and output surface of the ZIM. Fig. S2 presents the splitting behavior of an actual ZIM with ε_r = 0.0001, μ_r = 0.0001 with constant input waveguide thickness of 0.5*a*. In order to get the optimal transmission of the splitter, there should be two matched condition satisfied. First, the impedance of the ZIM of the free space should be matched, which is usually met. Second, the eigen modes of both of input and output of the splitter should be matched. As we mentioned above, both the amplitude and the phase are uniform on the input/output surfaces. Consequently, we can regard the two output waveguides as a single one with a width of the sum of two output waveguide. When the width of the output waveguide equals to the counterpart of the input waveguide, the eigen modes match each other, in which a perfect transmission is obtained, which is shown in Fig. S2(a). Furthermore, the splitting ratio is proportional to the width of the output waveguide when both the matching conditions are satisfied. Then, we keep the total width of output waveguide constant equal to the input waveguide and calculate the transmission of both the output waveguides of WG_2 and WG_3, which are displayed in Fig. S2(b), where one can see that it maintains a perfect splitting ability i.e. almost no reflection, the splitting ratio is proportional to the width of output waveguides.

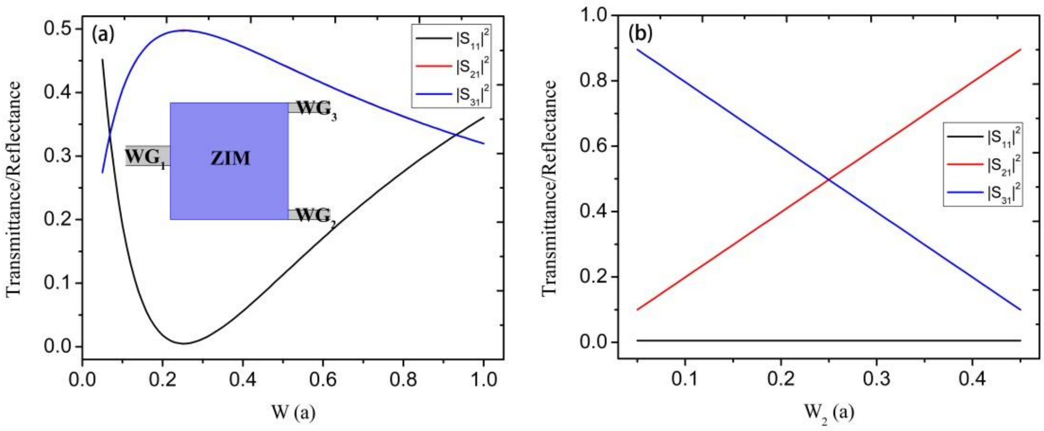

Figure S2. The splitting behavior of a beam splitter of an actual ZIM with ε_r = 0.0001, μ_r = 0.0001. (a) The transmittance /reflectance spectrums versus the width of output waveguides *W*, the width of the input waveguide is keep constant as 0.5*a*; (b) The transmittance/reflectance spectrums versus the width of output waveguide WG_2, the sum of the two output waveguide width is keep constant as 0.5*a* as well as the width of the input waveguide.

3. Angular flexibility of the beam splitters with ZIM

The ZIM we concerned is isotropic, the bending of the ZIM with a certain angle does not influence the behaviors of the beam splitters. Furthermore, the splitters are free from the introduction of a defect inside the ZIM. We also show such kind of ZIM beam splitter with a degree of flexibility such as introduction of defects and bending angles in Fig. S3.

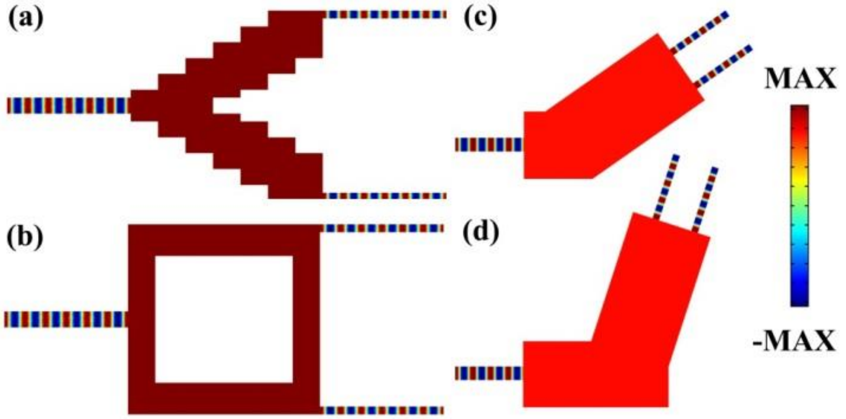

Figure S3. The electric field distributions of ZIM beam splitter with defects (a)-(b) and bending angle of 35° and 72° (c)-(d).

4. The eigen mode properties of the multiple unit cells of ZIPC at the zigzag edge

From Fig.4S, it evident that the output wave front of the ZIPC is planar, where the phase is uniform along the zigzag interface. However, the amplitude of the light field, which is indicated by E_z here, is position dependent. According to Bloch theorem, it is a periodic function with a period of the crystal constant of the photonic crystal. Fig. S5 shows the case where both the effective surface impedance and mode are perfectly matched. The input waveguide WG_1 with width of a located at the center of the entrance surface of PC, and the output waveguides WG_2/WG_3 with width of 0.5a located at the upper and underside of exit surface of PC. Noted that the perfect transmission (100%) also accomplished when the input/ output waveguides located at other matching position i.e. the same location of every period.

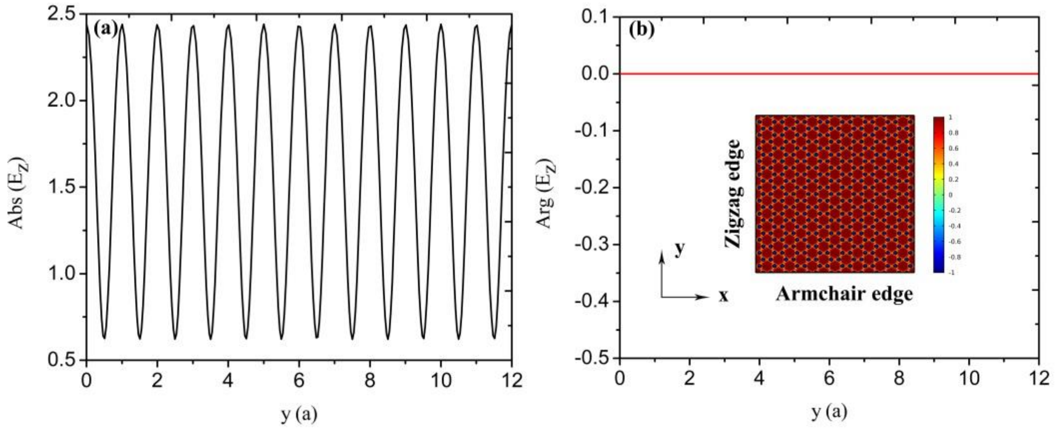

Figure S4. (a) Amplitude and (b) phase distributions along the zigzag edge of the exit surface of ZIPC. The inset shows the electric field distribution of the bulk photonic crystal with zigzag and armchair edges.

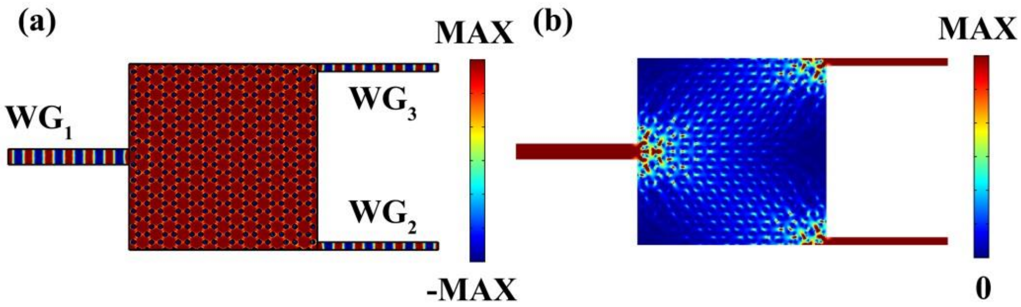

Figure S5. The representative example of ZIPC beam splitter with both mode and impedance perfectly matched. (a) The electric field distribution. (b) The corresponding energy flux density distribution.

Reference:

S1. Alù, A. Silveirinha, M. G. Salandrino, A. & Engheta, N. Epsilon-near-zero metamaterials and electromagnetic sources: Tailoring the radiation phase pattern. *Physical Review B* **75**, 155410 (2007).
S2. Hwang, R. B. Hsu, N. C. & Chin, C. Y. A spatial beam splitter consisting of a near-zero refractive index medium. *IEEE Transactions on Antennas and Propagation* **60**, 417-420 (2012).
